# Analysis of potential barriers for PrEP non-users among PrEP-eligible MSM in Germany

**DOI:** 10.1186/s12889-026-27039-3

**Published:** 2026-03-27

**Authors:** Helena Waldorf, Ulrich Marcus, Sara Iannuzzi, Stefan Albrecht, Jens Hoebel, Barbara Gunsenheimer-Bartmeyer, Viviane Bremer, Max von Kleist, Uwe Koppe

**Affiliations:** 1 Project Group 5 “Systems Medicine of Infectious Disease”, Robert Koch Institute, Berlin, Germany; 2 Department of Mathematics and Computer Science, Freie Universität Berlin, Berlin, Germany; 3 Department of Infectious Disease Epidemiology, Robert Koch Institute, Berlin, Germany; 4 Department of Epidemiology and Health Monitoring, Robert Koch Institute, Berlin, Germany

**Keywords:** HIV prevention, barriers, PrEP, TDF/FTC, MSM, uptake

## Abstract

**Background:**

Daily oral pre-exposure prophylaxis (PrEP) provides effective protection against HIV. Since September 2019, the costs of PrEP have been covered by statutory health insurance in Germany. While a considerable fraction of PrEP-eligible individuals receives PrEP, coverage is inhomogeneous across Germany. This study aims to identify potential barriers associated with PrEP non-use among PrEP-eligible MSM.

**Methods:**

Based on the PrApp online cross-sectional study, we analyzed 1,027 PrEP users and 431 PrEP non-users. A PrEP indication was assumed for cis-MSM with a diagnosis of a bacterial STI (syphilis, gonorrhea, chlamydia) or hepatitis C (12 months), ≥ 2 sex partners or sexualized drug use (6 months). Characteristics between PrEP users and PrEP non-users were compared descriptively and using multivariable logistic regression.

**Results:**

PrEP non-users were more likely to be aged 18–29 years old (*P *< 0.05, reference group: 30–39 years) and to engage in sexualized drug use (*P *< 0.001). The highest HIV-specialists density (*P *< 0.01, reference group: 6–9 HIV-specialists per 10,000 gay men) was associated with PrEP use. Persons with sexualized drug use were more likely to report daily PrEP use as a barrier (34.3% vs. 16.9%, adjusted *P *< 0.05). Fear of side effects (54.5%) was the most common barrier and might be more pronounced in PrEP non-users living in a federal state with a high HIV-specialists density (67.9% vs. 51.8%, adjusted *P *= 0.08). PrEP non-users living in a federal state with a low HIV-specialists density described not wanting to discuss their sex life with their doctor (29.5% vs. 16.0%, adjusted *P *= 0.08) as a reason for PrEP non-use.

**Conclusions:**

Our analyses indicated structural barriers to PrEP use in federal states with a low HIV-specialists density (physician density, non-anonymity). For those with theoretical PrEP access (high HIV-specialists density), fear of side effects could potentially be addressed by effective risk communication.

**Supplementary Information:**

The online version contains supplementary material available at 10.1186/s12889-026-27039-3.

## Introduction

HIV remains a relevant public health threat, with globally 1.3 million new infections, 39.9 million people living with HIV (PLHIV) and 630,000 HIV-related deaths in 2023 [1]. Men who have sex with men (MSM) denote the most HIV affected group in Germany, accounting for 54.5% (1,200) of the estimated 2,200 new HIV infections and for 58.9% (57,000) of the estimated 96,700 PLHIV in 2023 [2].

While antiretroviral drugs are successfully used for the management of an infection, immediate and successful antiretroviral treatment of PLHIV (TasP) reduces patients’ viral loads below the limit of detection and therefore prevents further sexual transmission [3]. Another important public health strategy is HIV pre-exposure prophylaxis (PrEP), where antivirals are taken before a potential HIV exposure. PrEP with daily oral emtricitabine/tenofovir disoproxil (FTC/TDF) was approved by the U.S. Food and Drug Administration (FDA) in 2012, and in 2016 by the European Medicines Agency (EMA) [4]. Clinical trials demonstrated that daily oral PrEP can effectively protect individuals at risk from an HIV infection [5, 6], and demonstrated prophylactic efficacy of oral PrEP in MSM, when taken on demand [7].

More recently, long-acting antiretrovirals, such as bimonthly cabotegravir, or 6-montly lenacapavir injections demonstrated HIV risk reductions comparable to those of oral PrEP in fully adherent MSM [8, 9]. While long-acting PrEP may be more convenient to use for some individuals struggling with daily adherence, to date, oral TDF/FTC PrEP is widely available as a generic [10]. In fact, over 99% of the estimated ≈ 8 million PrEP initiations worldwide are with oral TDF/XTC (TDF/FTC and TDF/3TC) [11].

Oral TDF/XTC-based PrEP is available in all European Union member states, but access varies considerably: For example, in France any primary care physician can prescribe PrEP as of 2021 and the costs of PrEP are covered by public health insurance [12]. In the Netherlands, PrEP can be prescribed by general practitioners, but individuals need to pay for the medicine. In Germany, PrEP can be prescribed by licensed specialists to those with substantial HIV risk. Since September 2019, the costs of PrEP have been covered by statutory health insurance (SHI) [13, 14].

The estimated number of PrEP users in Germany was 15,600–21,600 MSM by June 2020, albeit with large regional differences and unsatisfied needs in rural areas [15]. These regional differences were also reflected in the estimated PrEP coverage, which was 24% (95% CI: 24–25%) by the end of 2020 [16]. The projected number of PrEP users was ≈ 50,000 and PrEP coverage was estimated at 62% by December 2025 in Germany [16].

It has recently been postulated that expanding the possibility of PrEP prescription to primary care in 2021 may have led to an increase of PrEP initiations in France. However, according to a recent study this expansion did not imply major changes in the sociodemographic characteristics of PrEP users compared to before the expansion [12]. To increase PrEP coverage, it is thus necessary to understand financial, structural, societal and behavioral barriers that may explain why PrEP-eligible MSM are not taking PrEP. Reasons for not taking PrEP specifically among PrEP-eligible MSM have been previously analyzed in a limited number of studies [17, 18], but not in Germany as of now. In this work, we analyzed data from an anonymized online-survey among MSM PrEP-users and -non-users to identify potential barriers to PrEP uptake in PrEP non-users with a PrEP indication in Germany.

## Methods

### Study design

The PrApp study is a cross-sectional study on PrEP uptake among MSM in Germany [19]. Participants were recruited through MSM dating apps (Grindr, Planetromeo and Hornet), anonymous testing checkpoints and a community website (https://prepjetzt.de/). In detail, instant messages or banner advertisements were utilized, directing potential participants in Germany to an online questionnaire, as well as flyers with a link and a QR code, which were distributed in checkpoints. In addition, participants were encouraged to recruit peers. Furthermore, persons had to be at least 18 years old to participate in the survey. Participants had to confirm that they had understood the terms and conditions of the survey and that they agreed to participate in this survey. This consent was required to complete the anonymous online survey, which was provided using the VOXCO Acuity4Survey platform.

The survey was offered in multiple languages (German, English, French, Spanish, Dutch, Polish, Russian, Swedish, Arabic and Turkish) and included questions on sexual behavior, sexually transmitted infections (STIs), hepatitis C, and reasons for not using PrEP. Questions on hepatitis C were included in the survey, because PrEP users can be at increased risk for hepatitis C, which is reflected in the German PrEP guidelines recommending a HCV antibody screening every 6–12 months among PrEP users [20]. Eligible participants could answer the questionnaire, using their mobile phones or desktop computers and at the end of the survey they had the opportunity to enter a lottery drawing for gift certificates. Data from two survey waves (24.02.2020 – 19.05.2020 and 02.11.2020 – 07.01.2021) were used in this analysis.

### Participant selection

We included all cis-male participants who completed the survey and either were not using PrEP or were currently using PrEP. Cis-male was defined as having a male gender identity and the sex assigned at birth being male. Moreover, participants from the last survey wave were excluded if they self-reported participation in a previous wave to avoid repeated participation. Concerning the PrEP non-users, only those who confirmed being HIV-negative were included. In order to identify those with a substantial HIV risk (= PrEP indication) we used criteria based on the German-Austrian PrEP guidelines [20] to include PrEP users and non-users with a PrEP indication in the analysis. A PrEP indication for PrEP non-users existed if at least one of the following criteria applied: sexualized drug use in the last six months, bacterial STI (syphilis, gonorrhea or chlamydia) or hepatitis C diagnosis in the last twelve months, or two or more sexual partners in the last six months while simultaneously having a low condom use (0–50%). The same criteria, except for low condom use, were applied to PrEP users to ensure comparability. Low condom use was excluded from the criteria for PrEP indication for PrEP users since previous studies reported infrequent condom use among PrEP users [19, 21].

### Outcomes and covariates

The outcomes of this analysis were variables associated with PrEP non-uptake and reasons for not using PrEP among those with a PrEP indication.

A complete list of survey questions and answer options can be found in the Additional file 1: Appendix S1. Similar to other MSM online surveys the survey design mostly employed pre-determined categories, because remembering for example specific numbers of sex partners was deemed to be less reliable, especially among participants with higher partner numbers. For the analysis the categorization of variables was based on the available answer options in the PrApp survey and in some cases further grouping was applied. The grouping is summarized in Additional file 1: Appendix S2.

The categories “I do not know” and “Prefer not to say” were removed from the variables and therefore treated as missing values. In terms of demographic characteristics, age groups were identified as 18–29, 30–39, 40–49 and 50–80 years. Furthermore, country of origin was summarized as “Germany” and “Outside Germany”. School leaving qualification was defined as “No school leaving qualification”, “Secondary school qualification (class 8 to 9)”, “Secondary school qualification / O-Levels (class 10)”, “A-Levels (class 12 or 13)’. Satisfaction with sex life was grouped into “Content” (combining happy and very happy), “Discontent” (combining unhappy and very unhappy) and “Sex does not matter right now”. We grouped the number of anal or vaginal sex partners within the last six months into 0, 1, 2–3, 4–5, 6–10, 11–20 and >20. The frequency of condom use for anal or vaginal sex was categorized into 0%, 25%, 50%, 75% and >95%. Regarding the number of sexual encounters with anal or vaginal sex in the last month the categories were defined as 0, 1–4x, 5–8x, 9–12x, >12x. Gender of sex partners was categorized as male, female and non-binary with each category as an individual variable, allowing for multiple answers. Sexualized drug use in the last six months, payment for sex in the last six months and STI symptoms in the last 12 months had the categories “Yes” and “No”. Sexualized drug injection was categorized into “No”, “Yes, but not in the last 6 months”, “Yes, 1–3x in the last 6 months” and “Yes, more than 3x in the last 6 months”. Regarding having had positive test for bacterial STIs or hepatitis C in your life this had the categories “Yes” and “No” for syphilis, gonorrhea, chlamydia, hepatitis C and never having had a diagnosis. The number of bacterial STI and hepatitis C diagnosis in the last 12 months (separately for syphilis, gonorrhea, chlamydia, hepatitis C) was grouped into 0, 1 and ≥2.

**Fig. 1:**
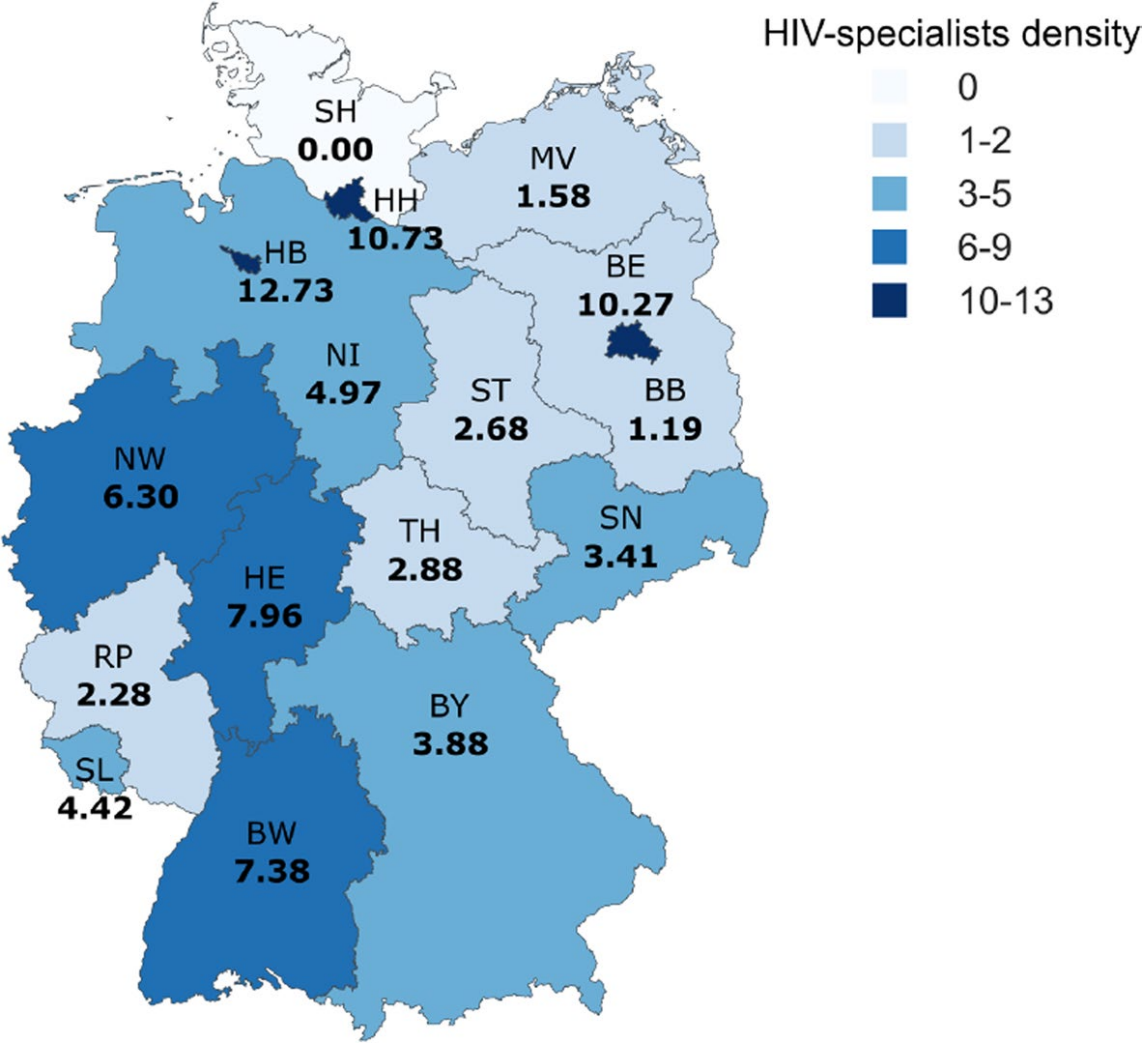
HIV-specialists density by federal state. The number of HIV specialists per 10,000 gay men is reported in bold. *BW* Baden-Württemberg, *BY* Bavaria, *BE* Berlin, *BB* Brandenburg, *HB* Bremen, *HH* Hamburg, *HE* Hesse, *MV* Mecklenburg Western Pomerania, *NI* Lower Saxony, *NW* North Rhine-Westphalia, *RP* Rhineland Palatinate, *SL* Saarland, *SN* Saxony, *ST* Saxony-Anhalt, *SH* Schleswig-Holstein, *TH* Thuringia

The federal states of residence were not directly included as a covariate but, encoded as the number of HIV-specialists per 10,000 gay men for each federal state and then grouped into 0, 1–2, 3–5, 6–9 and 10–13 as HIV-specialists density by federal state as described elsewhere [15]. The number of HIV-specialists is based on the website of the German association of physicians in private practice providing HIV-care “Deutsche Arbeitsgemeinschaft niedergelassener Ärzte in der Versorgung HIV-Infizierter e. V.” (dagnä) (30.06.2020) [22] and the estimate of the gay population in each federal state from the European MSM Internet Survey 2017 (EMIS) [23]. The highest HIV-specialists density could be found in the city states Berlin (10.27 HIV-specialists per 10,000 gay men), Hamburg (10.73 HIV-specialists per 10,000 gay men) and Bremen (12.73 HIV-specialists per 10,000 gay men), while the lowest HIV-specialists densities were observed in Mecklenburg Western Pomerania, Brandenburg and Schleswig-Holstein (Fig. 1). There was a tendency towards a lower HIV-specialists density in the east German federal states.

Moreover, the first three digits of the postcode were used to determine whether the participant lived in a rural or urban area based on data from the federal institute for research on building, urban affairs and spatial development “Bundesinstitut für Bau-, Stadt- und Raumforschung” (BBSR) [24]. The postcode was utilized to find the matching administrative seat in the BBSR data and then further spatial information was derived from this. For some postal codes, the matching administrative unit had to be determined manually, as an administrative seat included several postcodes.

Based on the monthly household net income the monthly net equivalent income was calculated by dividing the mean of the monthly household net income category by the square root of the number of persons in the household [25]. For the highest category the population-weighted median of persons with a household net income over 5,000€ was used, which was 6,260€ (SOEP 2018) [26]. The monthly net equivalent income was categorized into <1,000€, 1,000 – <2,000€, 2,000 – <3,000€, 3,000 – <4,000€, 4,000 – <5,000€, and ≥5,000€.

### Statistical methods

We calculated the probability of participation for the federal states and the Wilson confidence interval to ensure that there are no extreme differences between the federal states. We considered differences in the participation probability of >1% as a meaningful difference.

Categorical variables are shown as absolute numbers and proportions. In univariate analyses, differences in the distribution of reasons for not using PrEP were analyzed using the Pearson’s chi-squared test and for low frequencies with Fisher’s exact test with an alpha level of 0.05. The reasons for PrEP non-uptake were analyzed in relation to PrEP indication and HIV-specialist density. *P*-values were adjusted with the Benjamini-Hochberg procedure to correct for multiple testing.

For the multivariable analysis the variables were one-hot encoded, meaning each category was encoded as a new binary variable, except for the most frequent category which was used as reference to avoid multicollinearity. Furthermore, missing values were treated as a separate category. We employed a multivariable logistic regression model with elastic net regularization and tuned the hyper-parameters (mixing parameter λ and the regularization strength α). First the optimal regularization strength was determined by 15-fold stratified repeated cross-validation for a fixed mixing parameter λ ∈ [0.1, 0.2, 0.3, 0.4, 0.5, 0.6, 0.7, 0.8, 0.9, 1.0] and the optimal regularization strength was then chosen based on the elbow point (maximized AUC score and minimized number of non-zero coefficients). Next, for each mixing parameter λ a regression model was fitted with the selected optimal regularization strength. The model with the lowest number of non-zero coefficients and lowest Akaike-Information-Criterion (AIC) score was chosen. Finally, the final selected logistic regression model was used in a stratified bootstrap with 10,000 samples with replacement, resulting in the bootstrapped regression coefficients, which were then used to calculate the 95% percentile confidence interval and to determine the single sided bootstrap-based *P*-values.

## Results

### Participants

**Fig. 2:**
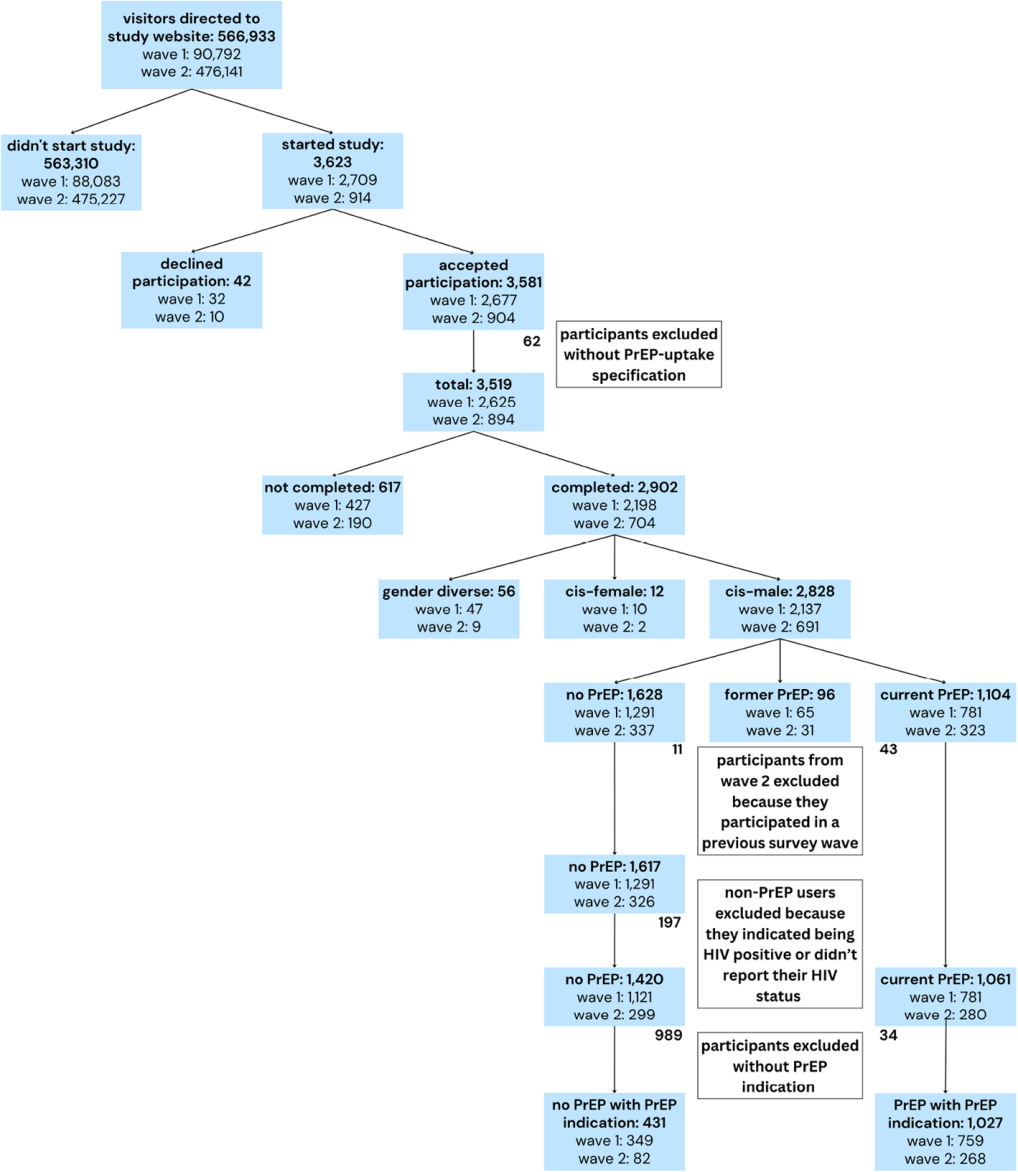
PrApp participant selection for analysis of factors associated with PrEP use. The two survey waves were conducted 24.02.2020 – 19.05.2020 and 02.11.2020 – 07.01.2021

Overall, 566,933 visitors were directed to the study website in the two survey waves, with 3623 starting the survey and 3581 accepting the participation. 62 participants, who did not specify their PrEP uptake, were excluded. The 3519 participants (first wave: 2625, second wave: 894) were further selected for study completion (2902) and study completion and being cis-male (2828). Of the latter, 1628 participants were not using PrEP and 1104 were currently using PrEP. 96 Participants who answered that they were former PrEP users were excluded from the analysis. 54 participants from the second wave, who self-reported having already participated in a previous wave, were identified and removed from analysis. Further, 197 PrEP non-users who did not confirm to be HIV-negative were excluded. Next, of the remaining 1420 PrEP non-users and 1061 current PrEP users only those who fulfilled the criteria for PrEP indication were included. In the end, there were 431 PrEP non-users with a PrEP indication and 1027 current PrEP users with a PrEP indication (Fig. 2).

To understand if the data is representative across Germany or has a spatial bias, we calculated the probability of participation in the PrApp study for each federal state based on the estimated gay population, as shown in Table 1. The participation probability ranged from 0.12% (Schleswig-Holstein) to 0.47% (Berlin), indicating a comparable probability of participation between the federal states. Importantly, the data includes participants from across Germany as opposed to only one or two federal states.

**Table 1:** Probability of participating in PrApp study by federal state

**Federal state**	Estimated gay population, n (EMIS 2017)	PrApp participants with PrEP indication, n	Probability of participation % (95% CI)
Baden-Württemberg	44651	115	0.26% (0.21–0.31%)
Bavaria	54061	212	0.39% (0.34–0.45%)
Berlin	59394	282	0.47% (0.42–0.53%)
Brandenburg	8395	20	0.24% (0.15–0.37%)
Bremen	3930	9	0.23% (0.12–0.43%)
Hamburg	17713	78	0.44% (0.35–0.55%)
Hesse	31422	134	0.43% (0.36–0.50%)
Mecklenburg Western Pomerania	6329	19	0.30% (0.19–0.47%)
Lower Saxony	28175	79	0.28% (0.23–0.35%)
North Rhine-Westphalia	79376	301	0.38% (0.34–0.42%)
Rhineland-Palatinate	13156	57	0.43% (0.33–0.56%)
Saarland	4520	12	0.27% (0.15–0.46%)
Saxony	20499	57	0.28% (0.21–0.36%)
Saxony-Anhalt	7454	15	0.20% (0.12–0.33%)
Schleswig-Holstein	13395	16	0.12% (0.07–0.19%)
Thuringia	6956	12	0.17% (0.10–0.30%)

*PrEP* pre-exposure prophylaxis, *CI* Wilson confidence interval

Current PrEP users had a median age of 38 years (IQR: 31–45), while PrEP non-users had a median age of 35 years (IQR: 28–43.5), (Table 2). The majority of the PrApp participants with a PrEP indication were born in Germany (81.2%).

**Table 2:** Baseline characteristics of PrApp participants with PrEP indication and the PrEP indication criteria, stratified by PrEP and PrEP non-users

	Participants, n (%)	PrEP users, n (%)	PrEP non-users, n (%)
Total (n)	1458	1027	431
Age (years)			
Median (IQR)	37 (30–45)	38 (31–45)	35 (28–43.5)
18–29, n (%)	337 (23.1%)	201 (19.6%)	136 (31.6%)
30–39, n (%)	490 (33.6%)	358 (34.9%)	132 (30.6%)
40–49, n (%)	403 (27.6%)	306 (29.8%)	97 (22.5%)
50–80, n (%)	228 (15.6%)	162 (15.8%)	66 (15.3%)
Missing	0 (0.0%)	0 (0.0%)	0 (0.0%)
Country of origin, n (%)			
Germany	1184 (81.2%)	847 (82.5%)	337 (78.2%)
Outside Germany	274 (18.8%)	180 (17.5%)	94 (21.8%)
Missing	0 (0.0%)	0 (0.0%)	0 (0.0%)
Monthly net equivalent income, n (%)			
<1000€	153 (10.5%)	79 (7.7%)	74 (17.2%)
1000 – <2000€	360 (24.7%)	234 (22.8%)	126 (29.2%)
2000 – <3000€	414 (28.4%)	294 (28.6%)	120 (27.8%)
3000 – <4000€	238 (16.3%)	189 (18.4%)	49 (11.4%)
4000 – <5000€	176 (12.1%)	143 (13.9%)	33 (7.7%)
≥5000€	54 (3.7%)	44 (4.3%)	10 (2.3%)
Missing	63 (4.3%)	44 (4.3%)	19 (4.4%)
HIV-specialists density, n (%)			
0	16 (1.1%)	12 (1.2%)	4 (0.9%)
1–2	123 (8.4%)	81 (7.9%)	42 (9.7%)
3–5	360 (24.7%)	255 (24.8%)	105 (24.4%)
6–9	550 (37.7%)	369 (35.9%)	181 (42.0%)
10–13	369 (25.3%)	288 (28.0%)	81 (18.8%)
Missing	40 (2.7%)	22 (2.1%)	18 (4.2%)
Condom use, n (%)			
0%	384 (26.3%)	322 (31.4%)	62 (14.4%)
25%	452 (31.0%)	339 (33.0%)	113 (26.2%)
50%	255 (17.5%)	154 (15.0%)	101 (23.4%)
75%	161 (11.0%)	106 (10.3%)	55 (12.8%)
>95%	183 (12.6%)	86 (8.4%)	97 (22.5%)
Missing	23 (1.6%)	20 (1.9%)	3 (0.7%)
Sex partners in the last 6 months, n (%)			
0	10 (0.7%)	2 (0.2%)	8 (1.9%)
1	24 (1.6%)	3 (0.3%)	21 (4.9%)
2–3	257 (17.6%)	123 (12.0%)	134 (31.1%)
4–5	265 (18.2%)	164 (16.0%)	101 (23.4%)
6–10	325 (22.3%)	242 (23.6%)	83 (19.3%)
11–20	228 (15.6%)	193 (18.8%)	35 (8.1%)
>20	333 (22.8%)	286 (27.8%)	47 (10.9%)
Missing	16 (1.1%)	14 (1.4%)	2 (0.5%)
Positive bacterial STI or hepatitis C diagnosis in the last 12 months, n (%)			
Yes	549 (37.7%)	413 (40.2%)	136 (31.6%)
No	324 (22.2%)	231 (22.5%)	93 (21.6%)
Missing	585 (40.1%)	383 (37.3%)	202 (46.9%)
Sexualized drug use in the last 6 months, n (%)			
Yes	331 (22.7%)	201 (19.6%)	130 (30.2%)
No	1097 (75.2%)	808 (78.7%)	289 (67.1%)
Missing	30 (2.1%)	18 (1.8%)	12 (2.8%)

*IQR* interquartile range, *PrEP* pre-exposure prophylaxis, *STI* sexually transmitted infection

Among PrEP non-users the most frequent monthly net equivalent category (29.2%) was an income of 1,000 – <2,000€, whereas it was 2,000 – <3,000€ (28.6%) for PrEP users. Of PrEP non-users, 22.5% reported very consistent condom use (>95% of sexual contacts) and 14.4% no use of any condoms, whereas 8.4% of PrEP users had very consistent condom use and 31.4% were not using condoms at all. Furthermore, 30.2% of PrEP non-users described sexualized drug use in the last six months vs. 19.6% for PrEP users. 28.0% of PrEP users were living in a federal state with a high HIV-specialists density, vs. 18.8% for PrEP non-users. Most PrEP users had >20 sex partners in the last six months (27.8%), whereas it was 2–3 sex partners (31.1%) for non-PrEP users.

The proportion of PrEP users among PrApp participants for each federal state varied between 0.40 (Saxony-Anhalt) and 0.89 (Bremen), depicted in Table 3.

**Table 3:** PrEP user proportion among PrApp participants with PrEP indication

**Federal state**	PrApp participants, n	PrEP users, n	PrEP user proportion
Baden-Württemberg	115	72	0.63
Bavaria	212	155	0.73
Berlin	282	218	0.77
Brandenburg	20	12	0.60
Bremen	9	8	0.89
Hamburg	78	62	0.79
Hesse	134	99	0.74
Mecklenburg Western Pomerania	19	14	0.74
Lower Saxony	79	56	0.71
North Rhine-Westphalia	301	198	0.66
Rhineland-Palatinate	57	43	0.75
Saarland	12	8	0.67
Saxony	57	36	0.63
Saxony-Anhalt	15	6	0.40
Schleswig-Holstein	16	12	0.75
Thuringia	12	6	0.50

*PrEP* pre-exposure prophylaxis

### Variables associated with not using PrEP

**Fig. 3:**
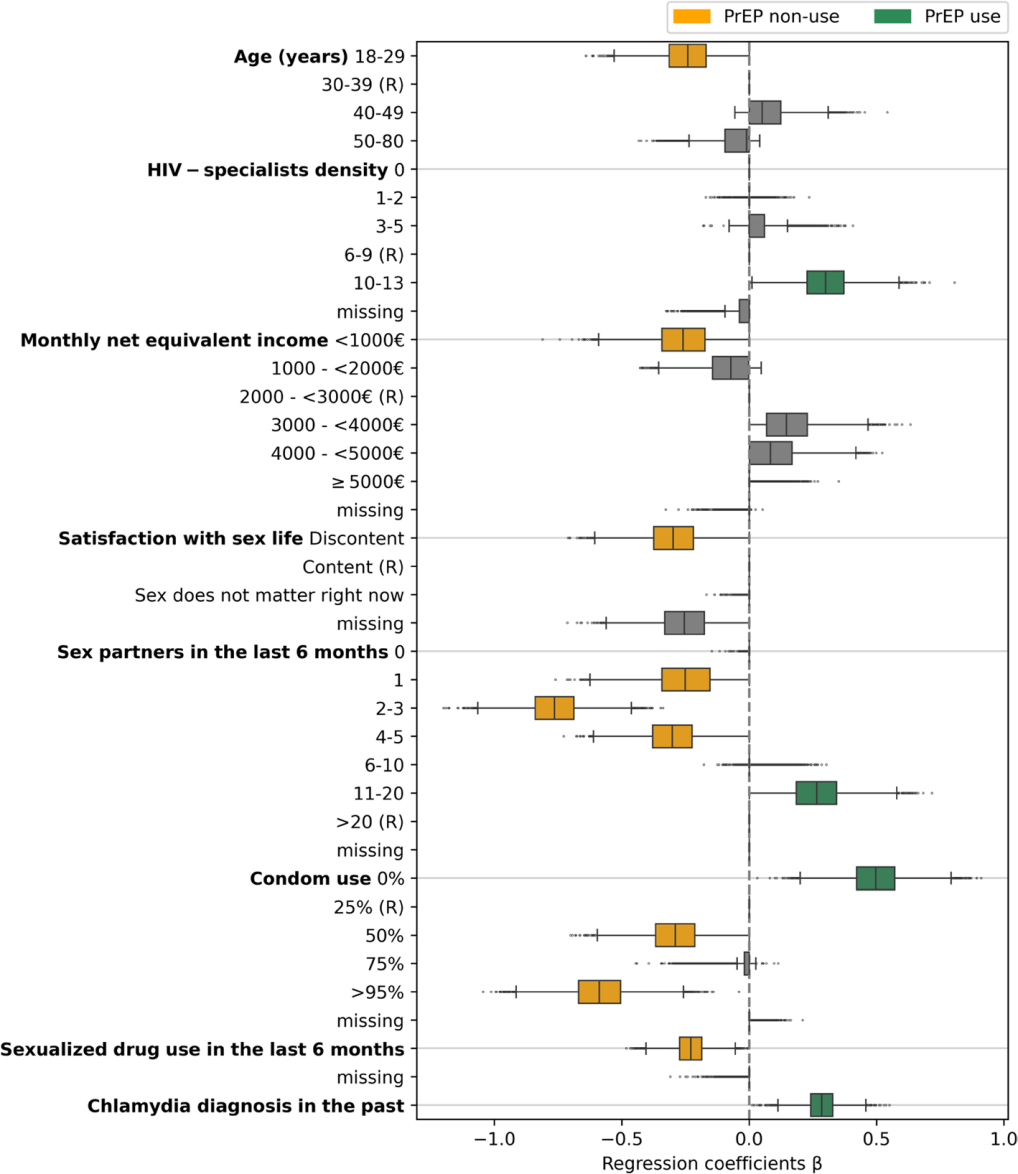
Distribution of bootstrapped regression coefficients for the significant variables (in orange and green) with *P*-value < 0.05 and their strata (in grey). (R) indicates the reference category, which is just depicted for completeness (no coefficients estimated). Significant “missing” categories are not highlighted (in green or orange), nor are they shown with their strata. For some categories, the interquartile ranges of bootstrapped coefficient estimates is zero with no boxplot depicted or only outliers as dots

The multivariable analysis resulted in 13 variables associated with using or not using PrEP (*P*-value < 0.05), which are depicted with their strata in Fig. 3. A complete list of all variables and their regression coefficients with corresponding 95% CIs and *P*-values are depicted in Additional file 1: Appendix S3. Treating missing values as separate categories in the analysis, led to three significant “missing” categories in addition to the 13 significant variables, which is a limitation of the missing value strategy.

Compared to the reference category >20 sex partners in the last six months, lower numbers of sex partners (1 sex partner: β = −0.27, 95% CI −0.51 – 0.00, *P* = 0.046; 2–3 sex partner: β = −0.77, 95% CI −0.98 – −0.54, *P* < 0.001; 4–5 sex partner: β = −0.31, 95% CI −0.52 – −0.08, *P *= 0.005), were associated with PrEP non-users, and 11–20 sex partners (β = 0.26, 95% CI 0.04 – 0.50, *P *= 0.012) were associated with PrEP users. Furthermore, sexualized drug use (SDU) in the last six months was more likely in PrEP non-users (β = −0.23, 95% CI −0.36 – −0.10, *P* < 0.001, reference group: no SDU). In relation to no chlamydia diagnosis in the past, having had a chlamydia diagnosis (β = 0.28, 95% CI 0.16 – 0.41, *P* < 0.001) was more pronounced in PrEP users. This could be explained by the fact that PrEP users are much more frequently screened for chlamydia than PrEP non-users because asymptomatic STI screening for PrEP users is covered. In comparison to the reference category (age: 30–39 years), PrEP non-users were more likely to be aged 18–29 (β = −0.24, 95% CI −0.45 – −0.03, *P* = 0.014) and also more frequently had female sexual partners (10.7% vs. 4.3%, *P* = 0.053, reference group: no female sex partners). Additionally, PrEP non-users were more likely discontent with their sex life (β = −0.31, 95% CI −0.52 – −0.07, *P* = 0.006), compared to the reference group being content with their sex life.

In terms of structural barriers a higher HIV-specialists density in the federal state of residence (10–13 HIV specialists per 10,000 gay men: β = 0.29, 95% CI 0.09 – 0.51, *P* = 0.004), contrasted to 6–9 HIV specialists per 10,000 gay men, was associated with PrEP use. Moreover, a low monthly net equivalent income of <1000€ (β = −0.27, 95% CI −0.50 – −0.01, *P* = 0.022, reference group: 2000 – <3000€) was associated with PrEP non-use.

### Reasons for not taking PrEP

To identify potential barriers to PrEP uptake, PrEP non-users with a PrEP indication were asked for their reasons for not taking PrEP, allowing multiple answers. The most common reason for not taking PrEP in PrEP non-users with a PrEP indication was the fear of side effects (54.5%), followed by the related effort of doctor visits and regular testing (34.3%) as depicted in Table 4.

The reasons for not taking PrEP were stratified by PrEP indication. We observed differences between PrEP non-users with a PrEP indication due to their number of sex partners combined with low condom use and/or bacterial STI/hepatitis C history and PrEP non-users with a PrEP indication due to sexualized drug use, but due to the effect sizes these were mostly not significant at the *P* < 0.05 level. In PrEP non-users with sexualized drug use, the necessity for daily intake of PrEP was identified as a barrier (34.3% vs. 16.9%, non-adjusted *P* = 0.002, adjusted *P* = 0.019), shown in Table 4.

**Table 4:** Reasons for not taking PrEP in PrEP non-users with PrEP indication, stratified by PrEP indication subgroups, multiple answers allowed

Reasons for not taking PrEP	All participants (*n* = 431)	Both (*n* = 63)	Sex partners and low condom use / STI / hepatitis C (*n* = 301)	Sexualized drug use (*n* = 67)	*P*-value^*a*^	Adjusted* P*-value^*b*^
Personal HIV risk too low	110 (25.5%)	13 (20.6%)	75 (24.9%)	22 (32.8%)	0.239	0.478
No doctor prescribing PrEP	88 (20.4%)	19 (30.2%)	55 (18.3%)	14 (20.9%)	0.746	0.994
Effort with doctor visits and regular tests	148 (34.3%)	24 (38.1%)	109 (36.2%)	15 (22.4%)	0.043	0.164
Not wanting to discuss sex life with doctor	116 (26.9%)	19 (30.2%)	83 (27.6%)	14 (20.9%)	0.333	0.532
Negative reactions of sexual partners / others	33 (7.7%)	7 (11.1%)	25 (8.3%)	1 (1.5%)	0.062	0.164
Fear of side effects	235 (54.5%)	27 (42.9%)	171 (56.8%)	37 (55.2%)	0.920	1.000
Daily pill too burdensome	89 (20.6%)	15 (23.8%)	51 (16.9%)	23 (34.3%)	0.002	0.019
Medical reasons	4 (0.9%)	0 (0%)	4 (1.3%)	0 (0%)	1.000	1.000

^*a*^Chi-squared test and for low frequencies with Fisher’s exact test, ^*b*^Benjamini-Hochberg for multiple testing correction. *PrEP* pre-exposure prophylaxis, *STI* sexually transmitted infection

Additionally, the reasons for not taking PrEP were stratified by the HIV-specialists density in the federal state of residence (Table 5). A low HIV-specialists density was defined as 0–9 HIV specialists per 10,000 gay men and a high HIV-specialists density referred to 10–13 HIV specialists per 10,000 gay men. The following describes the observed differences, although not significant at the *P* < 0.05 level. PrEP non-users living in a federal state with a low HIV-specialists density more frequently did not want to discuss their sex life with a doctor (29.5% vs. 16.0%, non-adjusted *P* = 0.021, adjusted *P* = 0.083). Moreover, PrEP non-users living in a federal state with a high HIV-specialists density might be associated with the barrier fear of side effects (67.9% vs. 51.8%, non-adjusted *P* = 0.013, adjusted *P* = 0.083).

**Table 5:** Reasons for not taking PrEP in PrEP non-users with PrEP indication, stratified by HIV-specialists density (Low: 0–9, High: 10–13), multiple answers allowed

Reasons for not taking PrEP	All participants (n = 431)	Low HIV-specialists density (n = 332)	High HIV-specialists density (n = 81)	*P*-value^*a*^	adjusted *P*-value^*b*^
Personal HIV risk too low	110 (25.5%)	80 (24.1%)	29 (35.8%)	0.045	0.121
No doctor prescribing PrEP	88 (20.4%)	70 (21.1%)	12 (14.8%)	0.266	0.306
Effort with doctor visits and regular tests	148 (34.3%)	122 (36.7%)	23 (28.4%)	0.200	0.306
Not wanting to discuss sex life with doctor	116 (26.9%)	98 (29.5%)	13 (16.0%)	0.021	0.083
Negative reactions of sexual partners / others	33 (7.7%)	27 (8.1%)	3 (3.7%)	0.255	0.306
Fear of side effects	235 (54.5%)	172 (51.8%)	55 (67.9%)	0.013	0.083
Daily pill too burdensome	89 (20.6%)	65 (19.6%)	21 (25.9%)	0.267	0.306
Medical reasons	4 (0.9%)	3 (0.9%)	1 (1.2%)	0.584	0.584

^*a*^Chi-squared test and for low frequencies with Fisher’s exact test, ^*b*^Benjamini-Hochberg for multiple testing correction. *PrEP* pre-exposure prophylaxis

## Discussion

We analyzed potential barriers to PrEP uptake by identifying differences between PrEP and PrEP non-users with a PrEP indication.

PrEP non-users more often had also sex with women (10.7% vs. 4.3%), but the association was weak and might have been a chance finding (*P* = 0.053). Nonetheless, studies have found that bisexual men are significantly less likely to have ever taken PrEP compared to gay men, as well as an association of experience with PrEP use and a gay sexual identity [27, 28]. Furthermore, studies show that bisexual men are also less likely to get tested for HIV [27, 29]. This may indicate that bisexual MSM are less likely to go to places that offer PrEP and HIV testing, e.g., checkpoints. One possible explanation for this could be that bisexual men do not perceive themselves as the target group for education on HIV prevention, as well as places offering related healthcare services, and rather tend to associate this with gay men. Consequently, a communication on HIV risks and prevention addressing bisexual men is important to reach all MSM in need of PrEP.

We found that among MSM with a PrEP indication, sexualized drug use was more common in PrEP non-users (*P* < 0.001). In support of our findings, reduced willingness to take PrEP was moderately associated with using methylenedioxy-methamphetamine (or ecstasy), amphetamine, marijuana, mephedrone, cocaine, heroin, gamma hydroxybutrate (GHB) or ketamine [30]. However, PrEP uptake has been frequently associated with sexualized drug use [31, 32]. Studies have identified different subgroups of MSM with substance use, which were associated with different risk behaviour [33–37]. Based on the available data we cannot differentiate between different substance use patterns and their context in our data. Future studies should focus on further contextualization. Similarly, it would be interesting to further explore the discrepancy between fear of PrEP side effects and substance use to further understand the relationship between side effects concerns and risk behaviour.

Our study indicating a lower PrEP uptake among young MSM (18–29 years; *P* = 0.014, reference group: 30–39 years) is supported by the findings of a meta-analysis on PrEP use among PrEP-eligible MSM, which found a higher percentage of current PrEP user in studies with a median age ≥ 30 years than in studies with a median age < 30 years (33.1% [95% CI 23.7–44.0; PI 12.9–62.2] vs. 16.3% [95% CI 10.9–23.6; PI 5.4–40.1], *P* = 0.0053) [38]. Young adults are at greater risk of HIV acquisition, with 43% of recent HIV infections among individuals of age 18–25 in Germany between 2008 and 2014 [39]. Consequently, it is important to develop effective public health strategies that target young MSM. Interestingly, a Dutch study from 2001 [40] found that young MSM are more likely to contract HIV from their main sex partner than from casual sex partner, indicating among other things the need for relationship-level HIV prevention strategies for young MSM. However, this finding dated to the time before TasP, and consequently the risk of contracting HIV have changed considerably, as well as perceived risks [41].

PrEP non-users were also more likely to have a low monthly net equivalent income of <1,000€ (*P* = 0.022, reference group: 2,000 – <3,000€). A study among young app-using MSM in California, as well as a retrospective study based on electronic medical records of federally-qualified health centers in New York, showed an association between PrEP use and higher income [42, 43]. Since September 2019, the costs of PrEP have been covered by statutory health insurance for individuals with substantial HIV risk. Due to the coverage, PrEP should be accessible independent of income, however, our findings suggest that the economic situation still plays a role. This is supported by a study on the changes of sociodemographic profiles of PrEP users before and after PrEP coverage conducted at a cross-sectoral sexual health centre in Germany, which showed no significant sociodemographic differences [44]. A potential reason could be that individuals with a lower net equivalent income are less aware that PrEP costs are covered by statutory health insurance, or they do not have the time and capacity to access PrEP. The former could be addressed by a targeted information campaign. Regarding the latter, as HIV-specialists are mostly located in urban areas, transportation to visit a PrEP prescriber is already time-consuming, which could be a barrier for MSM with a low income. By making PrEP more easily accessible, for example using telemedicine [45], PrEP uptake among low income MSM could potentially be facilitated. Telemedicine for PrEP may also address structural barriers due to low HIV-specialists density.

Concerning structural barriers, our analyses showed a significant association between PrEP uptake and a high HIV-specialists density (*P* = 0.004, reference group: 6–9 HIV-specialists per 10,000 gay men), suggesting that accessibility of PrEP plays an important factor for PrEP uptake. While the relationship between HIV-specialists density and PrEP use appears plausible and aligns with other published research [15], future investigations should use a causal modelling approach to eliminate possible confounding. Importantly, it is known that MSM with their place of residence in rural areas access PrEP in urban areas and also across federal states. Additionally, not only the distance to a doctor prescribing PrEP can be a barrier for not taking PrEP, but also the associated stigma and lack of anonymity for PrEP care in rural areas [46].

Overall, the most common reason for not taking PrEP (54.5%) was the fear of side effects. Concerns about side effects as a barrier to PrEP uptake have been found in multiple studies [47–50]. Notably, the fear of long-term side effects as a reason for PrEP discontinuation was identified in former PrEP users [51]. While TDF may decrease bone mineral density [52], a systematic review on oral PrEP with TDF/FTC or TDF detected no increased risk of bone fracture, and while they identified an association with a slightly elevated risk of gastrointestinal and renal adverse events, most adverse events were minor and reversible [53]. By educating individuals who are eligible to use PrEP on the advantages and disadvantages of PrEP, including potential side effects, the fear of side effects could be addressed and personal decisions balancing the benefits and potential disadvantages of oral PrEP may be better informed.

The next most common reason was the effort with doctor visits and regular testing with 34.3%. A recent study has demonstrated that online-mediated and 6-monthly PrEP monitoring are non-inferior to in-clinic and to 3-monthly monitoring [54], which could be an approach to reduce this barrier.

As a potential barrier we identified daily PrEP uptake for those PrEP non-users with a PrEP indication due to their sexualized drug use (34.3% vs. 16.9%, non-adjusted *P* = 0.002, adjusted *P* = 0.019). Notably, it is suggested that daily PrEP adherence can be effective and feasible for MSM reporting chemsex (defined as crystal meth, GHB/GBL or mephedrone immediately before or during sex) [55]. However, having an alternative to daily PrEP, such as on-demand PrEP could be beneficial for this subgroup.

The below discussed observed differences between PrEP non-users living in a federal state with a low HIV-specialists density and a high HIV-specialists density were not statistically significant at the *P* < 0.05 level.

PrEP non-users living in a federal state with a low HIV-specialists density reported more often not wanting to discuss their sex life with a doctor (29.5% vs. 16.0%, non-adjusted *P* = 0.021, adjusted *P* = 0.083) as barrier. This underlines the impact of the limited number health-care providers licensed to prescribe PrEP on PrEP uptake. To address this and to cover the PrEP needs of MSM not finding a PrEP prescriber due to distance and/or lack of privacy, making PrEP accessible through telemedicine [45] or an increase in PrEP prescribers might be a strategy to overcome these barriers which should be investigated in future studies.

In contrast, PrEP non-users living in federal states with a high HIV-specialists density described as a potential reason for not taking PrEP, the pronounced fear of side effects (67.9% vs. 51.8%, non-adjusted *P* = 0.013, adjusted *P* = 0.083). This suggests that the pure access PrEP might be not sufficient, but that the communication on side effects is potentially important to allow them to make an informed decision.

A strength of our study is that it includes current PrEP users as well as PrEP non-users. This allows us to identify barriers for PrEP non-users with a PrEP indication. Notably, a limitation is that this not a causal analysis, but an investigation into potential barriers to PrEP uptake among those with a PrEP indication. Identifying these barriers allows to gain a deeper understanding on why those advised to take PrEP are not using it in times when the health insurance covers the costs of PrEP. Similarly, it is unclear how representative this study is, especially for individuals not using online resources. This is not expected to be of great impact as the use of the internet and dating apps is widely spread among MSM [56]. Additionally, we have no data whether all MSM using dating apps are all equally likely to join the study.

In the survey design pre-determined categories were utilized in the possible answer options, and thus no continuous variable values were available for the analysis. Furthermore, all data is self-reported, which could potentially induce a response bias. However, many participants reported for example low condom use, sexualized drug use and high number of sexual partner, which suggests that potential response biases are minor. Additionally, the identification of repeated participation in the survey was done by self-report, which poses a limitation.

We defined the PrEP indication criteria to meet the PrEP eligibility criteria in the German guidelines. Notably, these might not reflect all individuals in need of PrEP such as persons planning condomless anal intercourse in the future. Consequently our analysis excluded 34 of 1,061 PrEP users, whose PrEP eligibility cannot be determined based on the available data set. Furthermore, the analysis resulted in three significant “missing” categories, which has no meaningful interpretation. The reasons were that we treated missing values as separate categories in the analysis. An analysis, where missing values were replaced by the reference category, yielded identical results apart from the fact that “missing values” were not significant anymore, see Additional file 1: Appendix S4.

## Conclusions

Overall, we found that the fear of side effects is still a persistent barrier to PrEP uptake. In the context of sexualized drug use, on-demand PrEP could be an alternative as the uptake of daily pill was perceived as a barrier. Furthermore, the distance to a PrEP prescriber, as well as lack of anonymity were more frequent reasons for not taking PrEP in federal states with a low HIV-specialists density, which could potentially be addressed by telemedicine, warranting further research. For PrEP non-users living in federal states with a high HIV-specialists density the fear of side effects may be approached by designing effective risk communication. In summary, PrEP-eligible MSM in Germany may face different barriers to PrEP uptake, which need to be further studied.

## Supplementary Information

Additional file 1. This file (.pdf) contains Appendix S1: Excerpt of survey questions from PrApp survey, Appendix S2: Grouping of variables, Appendix S3: Comparison of PrEP and PrEP non-users with a PrEP indication and Appendix S4: Sensitivity analysis of multivariable logistic regression model (missing value imputation).

## Data Availability

The survey data are not publicly available due to privacy or ethical restrictions but are available from the corresponding author on reasonable request.

## Abbreviations

PrEP: Pre-exposure prophylaxis
MSM: Men, who have sex with men
STI: Sexually transmitted infection
SDU: Sexualized drug use
PLHIV: People living with HIV
TasP: Treatment as prevention
FTC/TDF: Emtricitabine/tenofovir disoproxil
FDA: U.S. Food and Drug Administration
EMA: European Medicines Agency
TDF/XTC: TDF/FTC and TDF/3TC
SHI: Statutory health insurance
dagnä e. V.: Deutsche Arbeitsgemeinschaft niedergelassener Ärzte in der Versorgung HIV-Infizierter e. V.
EMIS: European MSM Internet Survey
BBSR: Bundesinstitut für Bau-, Stadt- und Raumforschung
AIC: Akaike-Information-Criterion
CI: (Wilson) confidence interval
IQR: Interquartile range
BW: Baden-Württemberg
BY: Bavaria
BE: Berlin
BB: Brandenburg
HB: Bremen, HH] Hamburg
HE: Hesse
MV: Mecklenburg Western Pomerania
NI: Lower Saxony
NW: North Rhine-Westphalia
RP: Rhineland Palatinate
SL: Saarland
SN: Saxony
ST: Saxony-Anhalt
SH: Schleswig-Holstein
TH: Thuringia
