# Supplementary material for: Analysis of potential barriers for PrEP non-users among PrEP-eligible MSM in Germany
Source: BMC Public Health. 2026 Mar 27;26:1170. doi: 10.1186/s12889-026-27039-3 (PMC13063581; doi:10.1186/s12889-026-27039-3)
Supplement: Supplementary file 1 — Additional file 1. This file (.pdf) contains Appendix S1: Excerpt of survey questions from PrApp survey, Appendix S2: Grouping of variables, Appendix S3: Comparison of PrEP and PrEP non-users with a PrEP indication and Appendix S4: Sensitivity analysis of multivariable logistic regression model (missing value imputation). [file 12889_2026_27039_MOESM1_ESM.pdf]

Appendix: Analysis of potential barriers for PrEP non-users among PrEP-eligible MSM in Germany

Overview

Appendix S1: Excerpt of survey questions from PrApp survey

Appendix S2: Grouping of variables

Appendix S3: Comparison of PrEP users and PrEP non-users with a PrEP indication

Appendix S4: Sensitivity analysis of multivariable logistic regression model (missing value imputation)

Appendix S1: Excerpt of survey questions from PrApp survey

Question	Answer options
**PrEP usage**	
Are you currently taking or have you ever taken preexposure prophylaxis (PrEP) against HIV?	• Yes, I am taking PrEP on a daily basis.<br>• Yes, I take it intermittently when I think I need it<br>• Yes, I used to take PrEP, but I permanently stopped<br>• No
**General information**	
Have you participated in this survey before? e.g. July–October 2018, April-May 2019 or March-May 2020 (only in last survey wave)	• Yes<br>• No<br>• I do not remember
How old are you?	
What gender do you identify with?	• Male<br>• Female<br>• Inter<br>• Trans male<br>• Trans female<br>• Non-binary
What gender were you assigned at birth?	• Male<br>• Female<br>• Intersexual
In which country were you born?	List of countries with translations
What are the first three digits of the postal code of your residence in Germany?<br><br>If you are not living in Germany, please indicate the first three digits of the postal code of the place where you are predominantly staying (e.g. hotel).	
What is the overall net income of the household you live in?<br><br>With income we mean: salaries/wages, non-wage incomes, retirement pension, unemployment/housing benefits, family allowances, etc. From this income, please subtract all taxes, operating expenses, and social security contributions	• 1 to less than 1000 €<br>• 1000 to less than 2000 €<br>• 2000 to less than 3000 €<br>• 3000 to less than 4000 €<br>• 4000 to less than 5000 €<br>• 5000 € and more
How many people are living in your household?	• I am living alone<br>• xxx persons
Did you graduate from school?	• Yes<br>• No<br>• Prefer not to say
What school leaving qualification do you have?	• Secondary school qualification (“Hauptschulabschluss”/ “Volksschulabschluss”) / class 8 to 9<br>• Secondary school qualification (“Realschulabschluss”) / O-Levels (“Mittlere Reife”) / class 10<br>• Grammar school qualification (“Gymnasium” / “EOS”) / A-Levels (“Abitur”) / class 12 or 13, / Technical college qualification (“Fachhochschulreife”)
**Sexual behaviour and risk behaviour**	
Are you having sex with... Select all that apply:	• Men<br>• Women<br>• Non-binary people
With how many different partners have you had anal/vaginal sex within the last 6 months?	• 0<br>• 1<br>• 2-3<br>• 4-5<br>• 6-10<br>• 11-20<br>• more than 20<br>• I do not know
How often have you had anal/vaginal sex in the last month?	• Not at all<br>• 1-4 times<br>• 5-8 times<br>• 9-12 times<br>• More than 12 times<br>• I do not know

Question	Answer options
Have you taken any substances in the last 6 months to have more intense or longer sex?<br><br>e.g. GHB/GBL, crystal meth, ketamine, cocaine, speed, bath salts, mephedrone, ecstasy/MDMA	• Yes<br>• No<br>• Prefer not to say
Did you inject these substances?	• Yes, but not within the last 6 months<br>• Yes, 1-3 times within the last 6 months<br>• Yes, more often than 3 times within the last 6 months<br>• No<br>• Prefer not to say
Were you paid for sex within the last 6 months?<br><br>By payment we mean the exchange of money, gifts, or favors for sex.	• Yes<br>• No<br>• Prefer not to say
How often do you use condoms for anal/vaginal sex in periods when you are taking PrEP?	• Always (in more than 95% of the times)<br>• Often (about 75% of the times)<br>• About half of the times (50%)<br>• Sometimes (about 25% of the times)<br>• Never<br>• I do not know
How happy are you with your sex life at the moment?	• Very happy<br>• Happy<br>• I'm not sure<br>• Unhappy<br>• Very unhappy<br>• Sex is not important for me at the moment
**Sexually transmitted infections**	
Have you had symptoms of a sexually transmitted infection in the last 12 months,<br><br>e.g. pain when urinating, discharge, itching/pain in the anal area?	• Yes<br>• No<br>• Prefer not to say
For which of the following have you tested positive? / have you ever been diagnosed with? Select all that apply:	• Syphilis<br>• Gonorrhea<br>• Chlamydia<br>• Hepatitis C<br>• None<br>• I do not remember
How often have you been diagnosed with syphilis in the last 12 months?	• 1-10<br>• More than 10 times
How often have you been diagnosed with gonorrhea in the last 12 months?	• 1-10<br>• More than 10 times
How often have you been diagnosed with chlamydia in the last 12 months?	• 1-10<br>• More than 10 times
How often have you been diagnosed with hepatitis C in the last 12 months?	• 1-10<br>• More than 10 times
**Reasons for non-PrEP use**	
Why are you not using PrEP? Select all that apply:	• My HIV risk is not high enough.<br>• I am HIV-positive.<br>• I cannot take PrEP due to medical reasons.<br>• I cannot find a doctor to prescribe PrEP to me.<br>• I do not want to talk about my sex life with my doctor.<br>• The effort of regularly visiting a doctor for refills and medical tests is too high for me.<br>• Taking a pill every day is too demanding for me.<br>• I am afraid of negative reactions from others/sex partners.<br>• I am afraid of side effects.

The survey waves were conducted 24.02.2020 - 19.05.2020 and 02.11.2020 - 07.01.2021.

Appendix S2: Grouping of variables

**Variable**	**Survey answer options**	**Grouping**
Age	Age (continuous)	18–29<br>30–39 (R)<br>40–49<br>50–80
Country of origin	List of countries with translations	Germany<br>Outside Germany (R)
Urban-rural area	First three digits of<br>postal code of residence	Urban area (R)<br>Rural area
HIV-specialists density<br>in federal state of residence	First three digits of<br>postal code of residence	0<br>1-2<br>3-5<br>6-9 (R)<br>10-13
School leaving<br>qualification	*School leaving qualification*<br>Class 8/9<br>Class 10<br>A-Levels (class 12/13)<br><br>*Did you graduate school?*<br>Yes<br>No<br>Prefer not to say	No school leaving certificate<br>Class 8/9<br>Class 10<br>A-Levels (class 12/13) (R)
Monthly net<br>equivalent income	*Monthly household net income*<br><1000€<br>1000 – <2000€<br>2000 – <3000€ (R)<br>3000 – <4000€<br>4000 – <5000€<br>≥5000€<br><br>*People living in household*<br>I am living alone<br>Household size (continuous)	<1000€<br>1000 – <2000€<br>2000 – <3000€ (R)<br>3000 – <4000€<br>4000 – <5000€<br>≥5000€
Satisfaction with sex life	Very happy<br>Happy<br>Unhappy<br>Very unhappy<br>I am not sure<br>Sex is not important to me at the moment	Content (R)<br>Discontent<br>Sex does not matter right now
Sex partners<br>in the last 6 months	0<br>1<br>2-3<br>4-5<br>6-10<br>11-20<br>>20<br>I do not know	0<br>1<br>2-3<br>4-5<br>6-10<br>11-20<br>>20 (R)
Condom use	Always (>95%)<br>Often (75%)<br>Half of the times (50%)<br>Sometimes (25%)<br>Never<br>I do not know	0%<br>25% (R)<br>50%<br>75%<br>>95%
Sexual encounters<br>with anal or vaginal sex<br>in the last month	0x<br>1-4x<br>5-8x<br>9-12x<br>>12x<br>I do not know	0x<br>1-4x (R)<br>5-8x<br>9-12x<br>>12x
Sexualized drug use<br>in the last 6 months	Yes<br>No<br>Prefer not to say	Yes<br>No (R)
Sexualized<br>drug injection	Yes, but 0x in the last 6 months<br>Yes, 1-3x in the last 6 months<br>Yes, >3x in the last 6 months<br>No<br>Prefer not to say	Yes, but 0x in the last 6 months<br>Yes, 1-3x in the last 6 months<br>Yes, >3x in the last 6 months<br>No (R)
Payment for sex<br>in the last 6 months	Yes<br>No<br>Prefer not to say	Yes<br>No (R)

**Variable**	**Survey answer options**	**Grouping**
Syphilis diagnoses in the last 12 months	0-10 (Continuous)<br>>10	0 (R)<br>1<br>≥2
Gonorrhea diagnoses in the last 12 months	0-10 (Continuous)<br>>10	0<br>1 (R)<br>≥2
Chlamydia diagnoses in the last 12 months	0-10 (Continuous)<br>>10	0<br>1 (R)<br>≥2
Hepatitis C diagnoses in the last 12 months	0-10 (Continuous)<br>>10	0 (R)<br>1
Sex with men	*Are you having sex with*<br>*(multiple answers possible)*<br>Men<br>Women<br>Non-binary people	Yes<br>No (R)
Sex with women	same as in previous row	Yes<br>No (R)
Sex with non-binary people	same as in previous row	Yes<br>No (R)
STI symptoms in the last 12 months	Yes<br>No<br>Prefer not to say	Yes<br>No (R)
Syphilis diagnoses in the past	*Diagnoses in the past*<br>*(multiple answers possible)*<br>Syphilis<br>Gonorrhea<br>Chlamydia<br>Hepatitis C<br>None<br>I do not remember	Yes<br>No (R)
Gonorrhea diagnoses in the past	same as in previous row	Yes<br>No (R)
Chlamydia diagnoses in the past	same as in previous row	Yes<br>No (R)
Hepatitis C diagnoses in the past	same as in previous row	Yes<br>No (R)
No STI or hepatitis C diagnoses in the past	same as in previous row	Yes<br>No (R)

If the grouping is based on multiple survey answers, the different survey questions are italicized; (R) indicates the reference category.

Appendix S3: Comparison of PrEP users and PrEP non-users with a PrEP indication

	**PrEP users, n (%)**	**PrEP non-users, n (%)**	**β (95% CI)[a]**	***P*-value[b]**
**Total (n)**	1027	431		
**Age (years)**				
Median (IQR)	38 (31 - 45)	35 (28 - 43.5)	-	
18–29, n (%)	201 (19.6%)	136 (31.6%)	-0.24 (-0.45 - -0.03)	0.014
30–39, n (%)	358 (34.9%)	132 (30.6%)	0	
40–49, n (%)	306 (29.8%)	97 (22.5%)	0.06 (0.00 - 0.26)	0.319
50–80, n (%)	162 (15.8%)	66 (15.3%)	-0.03 (-0.25 - 0.00)	0.464
Missing, n (%)	0 (0%)	0 (0%)	-	
**Country of origin, n (%)**				
Germany	847 (82.5%)	337 (78.2%)	0.00 (0.00 - 0.16)	0.678
Outside Germany	180 (17.5%)	94 (21.8%)	0	
Missing	0 (0%)	0 (0%)	-	
**Urban-rural area (based on postcode), n (%)**				
Urban area	900 (87.6%)	352 (81.7%)	0	
Rural area	105 (10.2%)	61 (14.2%)	0.00 (-0.22 - 0.00)	0.538
Missing	22 (2.1%)	18 (4.2%)	0.00 (-0.16 - 0.00)	0.620
**HIV-specialists density in federal state of residence, n (%)**				
0	12 (1.2%)	4 (0.9%)	0.00 (0.00 - 0.00)	1.000
1-2	81 (7.9%)	42 (9.7%)	0.00 (0.00 - 0.00)	0.979
3-5	255 (24.8%)	105 (24.4%)	0.00 (0.00 - 0.20)	0.554
6-9	369 (35.9%)	181 (42.0%)	0	
10-13	288 (28.0%)	81 (18.8%)	0.29 (0.09 - 0.51)	0.004
Missing	22 (2.1%)	18 (4.2%)	0.00 (-0.16 - 0.00)	0.620
**School leaving qualification, n (%)**				
No school leaving qualification	7 (0.7%)	9 (2.1%)	0.00 (0.00 - 0.00)	0.985
Class 8/9	49 (4.8%)	20 (4.6%)	0.00 (0.00 - 0.00)	0.995
Class 10	190 (18.5%)	104 (24.1%)	-0.10 (-0.31 - 0.00)	0.199
A-Levels (class 12 or 13)	776 (75.6%)	288 (66.8%)	0	
Missing	5 (0.5%)	10 (2.3%)	0.00 (-0.01 - 0.00)	0.969
**Monthly net equivalent income, n (%)**				
<1000€	79 (7.7%)	74 (17.2%)	-0.27 (-0.50 - -0.01)	0.022
1000 – <2000€	234 (22.8%)	126 (29.2%)	-0.08 (-0.28 - 0.00)	0.245
2000 – <3000€	294 (28.6%)	120 (27.8%)	0	
3000 – <4000€	189 (18.4%)	49 (11.4%)	0.16 (0.00 - 0.39)	0.100
4000 – <5000€	143 (13.9%)	33 (7.7%)	0.09 (0.00 - 0.33)	0.246
≥5000€	44 (4.3%)	10 (2.3%)	0.00 (0.00 - 0.09)	0.895
Missing	44 (4.3%)	19 (4.4%)	0.00 (-0.04 - 0.00)	0.959
**Satisfaction with sex life, n (%)**				
Content	717 (69.8%)	206 (47.8%)	0	
Discontent	148 (14.4%)	108 (25.1%)	-0.31 (-0.52 - -0.07)	0.006
Sex does not matter right now	12 (1.2%)	14 (3.2%)	0.00 (0.00 - 0.00)	0.991
Missing	150 (14.6%)	103 (23.9%)	-0.26 (-0.47 - -0.02)	0.015
**Sex partners in the last 6 months, n (%)**				
0	2 (0.2%)	8 (1.9%)	0.00 (0.00 - 0.00)	0.995
1	3 (0.3%)	21 (4.9%)	-0.27 (-0.51 - 0.00)	0.046
2-3	123 (12.0%)	134 (31.1%)	-0.77 (-0.98 - -0.54)	<0.001
4-5	164 (16.0%)	101 (23.4%)	-0.31 (-0.52 - -0.08)	0.005
6-10	242 (23.6%)	83 (19.3%)	0.00 (0.00 - 0.12)	0.813
11-20	193 (18.8%)	35 (8.1%)	0.26 (0.04 - 0.50)	0.012
>20	286 (27.8%)	47 (10.9%)	0	
Missing	14 (1.4%)	2 (0.5%)	0.00 (0.00 - 0.00)	1.000
**Condom use, n (%)**				
0%	322 (31.4%)	62 (14.4%)	0.50 (0.28 - 0.71)	<0.001
25%	339 (33.0%)	113 (26.2%)	0	
50%	154 (15.0%)	101 (23.4%)	-0.29 (-0.51 - -0.07)	0.006
75%	106 (10.3%)	55 (12.8%)	0.00 (-0.18 - 0.00)	0.703
>95%	86 (8.4%)	97 (22.5%)	-0.59 (-0.83 - -0.35)	<0.001
Missing	20 (1.9%)	3 (0.7%)	0.00 (0.00 - 0.00)	0.975
**Sexual encounters with anal or vaginal sex in the last month, n (%)**				
0	69 (6.7%)	63 (14.6%)	-0.07 (-0.31 - 0.00)	0.316
1-4	442 (43.0%)	217 (50.3%)	0	
5-8	232 (22.6%)	68 (15.8%)	0.01 (0.00 - 0.23)	0.484
9-12	104 (10.1%)	40 (9.3%)	0.00 (-0.10 - 0.00)	0.876
>12	168 (16.4%)	38 (8.8%)	0.12 (0.00 - 0.36)	0.149
Missing	12 (1.2%)	5 (1.2%)	0.00 (0.00 - 0.00)	1.000
**Sexualized drug use in the last 6 months, n (%)**				
Yes	201 (19.6%)	130 (30.2%)	-0.23 (-0.36 - -0.10)	<0.001
No	808 (78.7%)	289 (67.1%)	0	
Missing	18 (1.8%)	12 (2.8%)	0.00 (-0.03 - 0.00)	0.963
**Sexualized drug injection, n (%)**				
Yes, 0x in the last 6 months	2 (0.2%)	5 (1.2%)	0.00 (0.00 - 0.00)	1.000

	PrEP users, n (%)	PrEP non-users, n (%)	β (95% CI)[a]	*P*-value[b]
Yes, 1-3x in the last 6 months	10 (1.0%)	4 (0.9%)	0.00 (0.00 - 0.00)	1.000
Yes, >3x in the last 6 months	17 (1.7%)	6 (1.4%)	0.00 (0.00 - 0.00)	1.000
No	171 (16.7%)	114 (26.5%)	0	
Missing	827 (80.5%)	302 (70.1%)	0.19 (0.06 - 0.31)	0.002
**Payment for sex in the last 6 months, n (%)**				
Yes	58 (5.6%)	37 (8.6%)	0.00 (-0.19 - 0.00)	0.677
No	964 (93.9%)	381 (88.4%)	0	
Missing	5 (0.5%)	13 (3.0%)	0.00 (-0.07 - 0.00)	0.919
**Syphilis diagnosis in the last 12 months, n (%)**				
0	166 (16.2%)	52 (12.1%)	0	
1	107 (10.4%)	42 (9.7%)	0.00 (-0.06 - 0.00)	0.940
≥2	11 (1.1%)	2 (0.5%)	0.00 (0.00 - 0.00)	1.000
Missing	743 (72.3%)	335 (77.7%)	0.00 (-0.08 - 0.01)	0.836
**Gonorrhoea diagnosis in the last 12 months, n (%)**				
0	173 (16.8%)	65 (15.1%)	0.00 (-0.20 - 0.00)	0.616
1	200 (19.5%)	56 (13.0%)	0	
≥2	68 (6.6%)	10 (2.3%)	0.00 (0.00 - 0.13)	0.804
Missing	586 (57.1%)	300 (69.6%)	-0.03 (-0.15 - 0.00)	0.239
**Chlamydia diagnosis in the last 12 months, n (%)**				
0	132 (12.9%)	40 (9.3%)	0.00 (-0.04 - 0.00)	0.949
1	221 (21.5%)	59 (13.7%)	0	
≥2	59 (5.7%)	2 (0.5%)	0.09 (0.00 - 0.27)	0.208
Missing	615 (59.9%)	330 (76.6%)	-0.13 (-0.26 - -0.01)	0.016
**Hepatitis C diagnosis in the last 12 months, n (%)**				
0	20 (1.9%)	17 (3.9%)	0	
1	5 (0.5%)	1 (0.2%)	0.00 (0.00 - 0.00)	1.000
Missing	1002 (97.6%)	413 (95.8%)	0.00 (0.00 - 0.15)	0.683
**Gender of sex partners, n (%) (multiple responses possible)**				
Male	1027 (100.0%)	430 (99.8%)	0.00 (0.00 - 0.00)	1.000
Female	44 (4.3%)	46 (10.7%)	-0.24 (-0.49 - 0.00)	0.053
Non-binary	33 (3.2%)	13 (3.0%)	0.00 (0.00 - 0.00)	0.999
Missing	0 (0%)	0 (0%)	-	
**STI symptoms in the last 12 months, n (%)**				
Yes	352 (34.3%)	135 (31.3%)	0.00 (-0.14 - 0.01)	0.741
No	673 (65.5%)	294 (68.2%)	0	
Missing	2 (0.2%)	2 (0.5%)	0.00 (0.00 - 0.00)	1.000
**Positive test in the past, n (%)**				
Syphilis	288 (28.0%)	99 (23.0%)	0.00 (-0.02 - 0.07)	0.860
Gonorrhoea	444 (43.2%)	131 (30.4%)	0.08 (0.00 - 0.20)	0.114
Chlamydia	420 (40.9%)	101 (23.4%)	0.28 (0.16 - 0.41)	<0.001
Hepatitis C	25 (2.4%)	18 (4.2%)	0.00 (-0.15 - 0.00)	0.684
Never	322 (31.4%)	177 (41.1%)	0.00 (0.00 - 0.10)	0.839

[a]multivariable logistic regression coefficients and bootstrap-based 95% percentile confidence interval, 0 indicates the reference category (no coefficients estimated), missing values were included as a separate category, [b]single sided bootstrap-based *P*-values. *CI* confidence interval, *PrEP* pre-exposure prophylaxis, *STI* sexually transmitted infection

Appendix S4: Sensitivity analysis of multivariable logistic regression model (missing value imputation)

	**PrEP users, n (%)**	**Non-PrEP users, n (%)**	**β (95% CI)[a]**	***P*-value[b]**
**Total (n)**	1027	431		
**Age (years)**				
Median (IQR)	38 (31 - 45)	35 (28 - 43.5)	-	
18–29, n (%)	201 (19.6%)	136 (31.6%)	-0.24 (-0.46 - -0.03)	0.013
30–39, n (%)	358 (34.9%)	132 (30.6%)	0	
40–49, n (%)	306 (29.8%)	97 (22.5%)	0.07 (0.00 - 0.27)	0.308
50–80, n (%)	162 (15.8%)	66 (15.3%)	-0.01 (-0.23 - 0.00)	0.502
Missing, n (%)	0 (0%)	0 (0%)	-	
**Country of origin, n (%)**				
Germany	847 (82.5%)	337 (78.2%)	0.00 (0.00 - 0.18)	0.617
Outside Germany	180 (17.5%)	94 (21.8%)	0	
Missing	0 (0%)	0 (0%)	-	
**Urban-rural area (based on postcode), n (%)**				
Urban area	900 (87.6%)	352 (81.7%)	0	
Rural area	105 (10.2%)	61 (14.2%)	0.00 (-0.21 - 0.00)	0.575
Missing	22 (2.1%)	18 (4.2%)	-	
**HIV-specialists density in federal state of residence, n (%)**				
0	12 (1.2%)	4 (0.9%)	0.00 (0.00 - 0.00)	1.000
1-2	81 (7.9%)	42 (9.7%)	0.00 (0.00 - 0.00)	0.976
3-5	255 (24.8%)	105 (24.4%)	0.00 (0.00 - 0.21)	0.518
6-9	369 (35.9%)	181 (42.0%)	0	
10-13	288 (28.0%)	81 (18.8%)	0.29 (0.09 - 0.51)	0.004
Missing	22 (2.1%)	18 (4.2%)	-	
**School leaving qualification, n (%)**				
No school leaving qualification	7 (0.7%)	9 (2.1%)	0.00 (0.00 - 0.00)	0.985
Class 8/9	49 (4.8%)	20 (4.6%)	0.00 (0.00 - 0.00)	0.994
Class 10	190 (18.5%)	104 (24.1%)	-0.11 (-0.33 - 0.00)	0.159
A-Levels (class 12 or 13)	776 (75.6%)	288 (66.8%)	0	
Missing	5 (0.5%)	10 (2.3%)	-	
**Monthly net equivalent income, n (%)**				
<1000€	79 (7.7%)	74 (17.2%)	-0.30 (-0.52 - -0.03)	0.014
1000 – <2000€	234 (22.8%)	126 (29.2%)	-0.09 (-0.28 - 0.00)	0.225
2000 – <3000€	294 (28.6%)	120 (27.8%)	0	
3000 – <4000€	189 (18.4%)	49 (11.4%)	0.15 (0.00 - 0.39)	0.103
4000 – <5000€	143 (13.9%)	33 (7.7%)	0.09 (0.00 - 0.33)	0.232
≥5000€	44 (4.3%)	10 (2.3%)	0.00 (0.00 - 0.09)	0.885
Missing	44 (4.3%)	19 (4.4%)	-	
**Satisfaction with sex life, n (%)**				
Content	717 (69.8%)	206 (47.8%)	0	
Discontent	148 (14.4%)	108 (25.1%)	-0.25 (-0.47 - -0.02)	0.018
Sex does not matter right now	12 (1.2%)	14 (3.2%)	0.00 (0.00 - 0.00)	0.993
Missing	150 (14.6%)	103 (23.9%)	-	
**Sex partners in the last 6 months, n (%)**				
0	2 (0.2%)	8 (1.9%)	0.00 (0.00 - 0.00)	0.996
1	3 (0.3%)	21 (4.9%)	-0.27 (-0.52 - 0.00)	0.042
2-3	123 (12.0%)	134 (31.1%)	-0.78 (-0.99 - -0.55)	<0.001
4-5	164 (16.0%)	101 (23.4%)	-0.32 (-0.53 - -0.08)	0.004
6-10	242 (23.6%)	83 (19.3%)	0.00 (0.00 - 0.11)	0.831
11-20	193 (18.8%)	35 (8.1%)	0.27 (0.04 - 0.50)	0.011
>20	286 (27.8%)	47 (10.9%)	0	
Missing	14 (1.4%)	2 (0.5%)	-	
**Condom use, n (%)**				
0%	322 (31.4%)	62 (14.4%)	0.50 (0.29 - 0.71)	<0.001
25%	339 (33.0%)	113 (26.2%)	0	
50%	154 (15.0%)	101 (23.4%)	-0.29 (-0.51 - -0.07)	0.006
75%	106 (10.3%)	55 (12.8%)	0.00 (-0.19 - 0.00)	0.695
>95%	86 (8.4%)	97 (22.5%)	-0.59 (-0.83 - -0.35)	<0.001
Missing	20 (1.9%)	3 (0.7%)	-	
**Sexual encounters with anal or vaginal sex in the last month, n (%)**				
0	69 (6.7%)	63 (14.6%)	-0.08 (-0.32 - 0.00)	0.293
1-4	442 (43.0%)	217 (50.3%)	0	
5-8	232 (22.6%)	68 (15.8%)	0.03 (0.00 - 0.25)	0.427
9-12	104 (10.1%)	40 (9.3%)	0.00 (-0.09 - 0.00)	0.892
>12	168 (16.4%)	38 (8.8%)	0.14 (0.00 - 0.38)	0.110
Missing	12 (1.2%)	5 (1.2%)	-	
**Sexualized drug use in the last 6 months, n (%)**				
Yes	201 (19.6%)	130 (30.2%)	-0.38 (-0.58 - -0.17)	<0.001
No	808 (78.7%)	289 (67.1%)	0	
Missing	18 (1.8%)	12 (2.8%)	-	
**Sexualized drug injection, n (%)**				

	PrEP users, n (%)	Non-PrEP users, n (%)	β (95% CI)[a]	*P*-value[b]
Yes, but 0x in the last 6 months	2 (0.2%)	5 (1.2%)	0.00 (0.00 - 0.00)	1.000
Yes, 1-3x in the last 6 months	10 (1.0%)	4 (0.9%)	0.00 (0.00 - 0.00)	1.000
Yes, >3x in the last 6 months	17 (1.7%)	6 (1.4%)	0.00 (0.00 - 0.00)	1.000
No	171 (16.7%)	114 (26.5%)	0	
Missing	827 (80.5%)	302 (70.1%)	-	
**Payment for sex in the last 6 months, n (%)**				
Yes	58 (5.6%)	37 (8.6%)	0.00 (-0.20 - 0.00)	0.653
No	964 (93.9%)	381 (88.4%)	0	
Missing	5 (0.5%)	13 (3.0%)	-	
**Syphilis diagnosis in the last 12 months, n (%)**				
0	166 (16.2%)	52 (12.1%)	0	
1	107 (10.4%)	42 (9.7%)	0.00 (-0.06 - 0.00)	0.935
≥2	11 (1.1%)	2 (0.5%)	0.00 (0.00 - 0.00)	1.000
Missing	743 (72.3%)	335 (77.7%)	-	
**Gonorrhoea diagnosis in the last 12 months, n (%)**				
0	173 (16.8%)	65 (15.1%)	0.00 (-0.18 - 0.00)	0.663
1	200 (19.5%)	56 (13.0%)	0	
≥2	68 (6.6%)	10 (2.3%)	0.00 (0.00 - 0.13)	0.800
Missing	586 (57.1%)	300 (69.6%)	-	
**Chlamydia diagnosis in the last 12 months, n (%)**				
0	132 (12.9%)	40 (9.3%)	0.00 (-0.03 - 0.00)	0.956
1	221 (21.5%)	59 (13.7%)	0	
≥2	59 (5.7%)	2 (0.5%)	0.10 (0.00 - 0.28)	0.180
Missing	615 (59.9%)	330 (76.6%)	-	
**Hepatitis C diagnosis in the last 12 months, n (%)**				
0	20 (1.9%)	17 (3.9%)	0	
1	5 (0.5%)	1 (0.2%)	0.00 (0.00 - 0.00)	1.000
Missing	1002 (97.6%)	413 (95.8%)	-	
**Gender of sex partners, n (%) (multiple responses possible)**				
Male	1027 (100.0%)	430 (99.8%)	0.00 (0.00 - 0.00)	1.000
Female	44 (4.3%)	46 (10.7%)	-0.23 (-0.48 - 0.00)	0.056
Non-binary	33 (3.2%)	13 (3.0%)	0.00 (0.00 - 0.00)	0.999
Missing	0 (0%)	0 (0%)	-	
**STI symptoms in the last 12 months, n (%)**				
Yes	352 (34.3%)	135 (31.3%)	0.00 (-0.14 - 0.01)	0.726
No	673 (65.5%)	294 (68.2%)	0	
Missing	2 (0.2%)	2 (0.5%)	-	
**Positive test in the past , n (%)**				
Syphilis	288 (28.0%)	99 (23.0%)	0.00 (-0.03 - 0.12)	0.813
Gonorrhoea	444 (43.2%)	131 (30.4%)	0.11 (0.00 - 0.32)	0.100
Chlamydia	420 (40.9%)	101 (23.4%)	0.41 (0.20 - 0.62)	<0.001
Hepatitis C	25 (2.4%)	18 (4.2%)	0.00 (-0.21 - 0.00)	0.680
Never	322 (31.4%)	177 (41.1%)	0.00 (0.00 - 0.08)	0.883

[a]multivariable logistic regression coefficients and bootstrap-based 95% percentile confidence interval, 0 indicates the reference category (no coefficients estimated), missing values were imputed with the reference category in the analysis, [b]single sided bootstrap-based *P*-values. *CI* confidence interval, *PrEP* pre-exposure prophylaxis, *STI* sexually transmitted infection
